# Supplementary material for: A New Systemic Disease Mouse Model for Glioblastoma Capable of Single-Tumour-Cell Detection
Source: Cells. 2024 Jan 19;13(2):192. doi: 10.3390/cells13020192 (PMC10814551; doi:10.3390/cells13020192)
Supplement: Supplementary file 1 [file cells-13-00192-s001.zip › cells-2774289-supplementary.pdf]

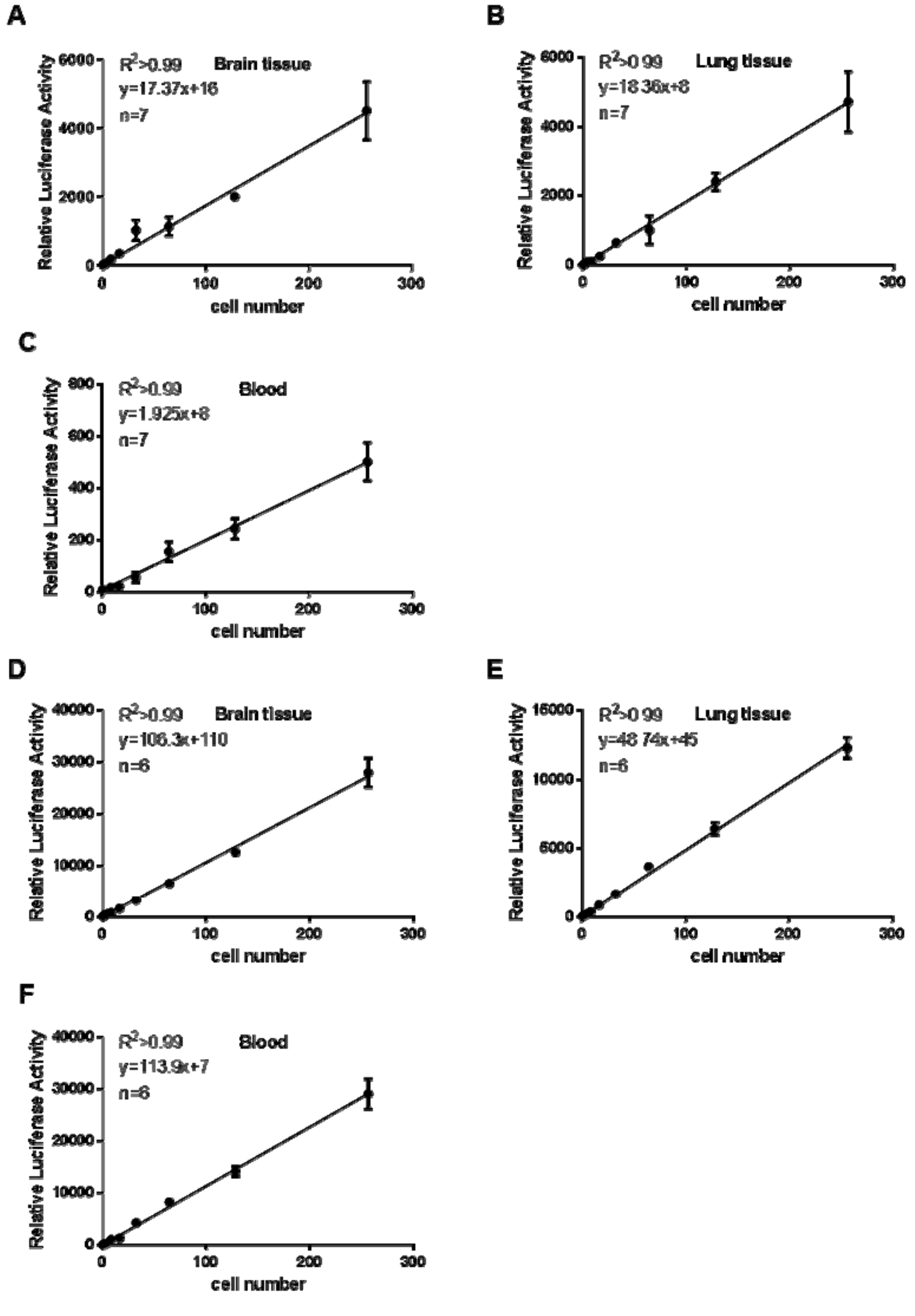

Figure S1. Standard curves for tumour cell quantitation for MU20-Fluc and U87MG-Gluc tumour cells. MU20-Fluc (A-C) and U87MG-Gluc (D-F) tumour cells were serially diluted to single cells and spiked with brain tissue (A and D), lung tissue (B and E) or blood (C and F). Cells were lysed and relative luciferase activity (R.L.U) measured. Cell quantitation was determined by best-fit linear regression analysis. Serial titrations were performed in 5mg tissue or 5μL blood for MU20-Fluc tumour cells (A-C), n=7, and 10mg tissue or 5μL blood for U87MG-Gluc tumour cells (D-F), n=6. Data is mean ± SEM.

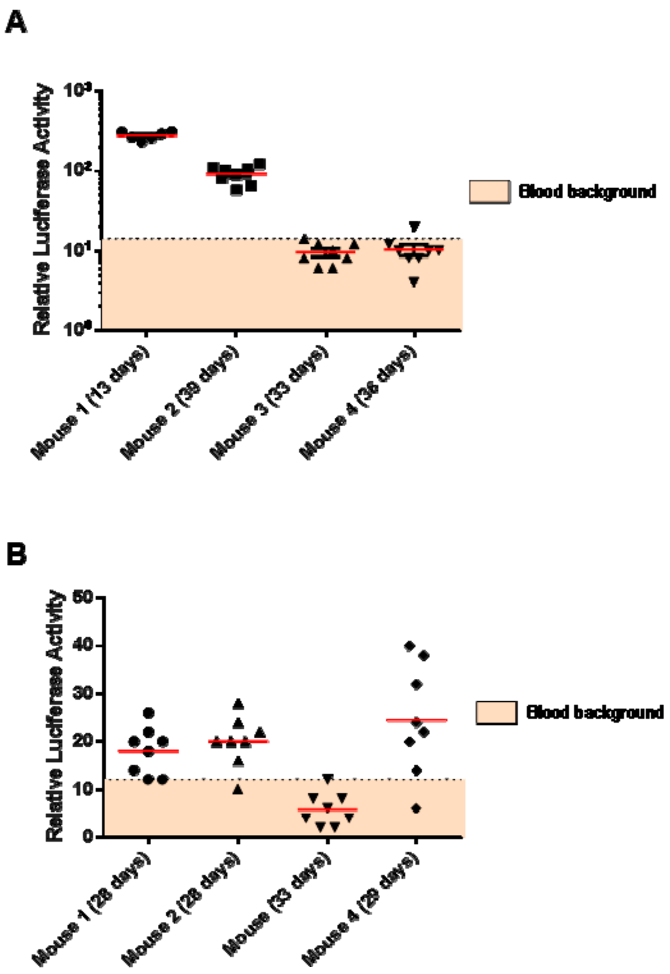

Figure S2. Circulating tumour cell detection varies between mice and over time. Mice were implanted with U87MG-Fluc (A) or MU20-Fluc (B) glioblastoma tumors. Following euthanasia, blood was collected from the jugular vein and analysed for luciferase activity in 5μL aliquots. Data represents the mean of n=8 independent samples per mouse (n=4).

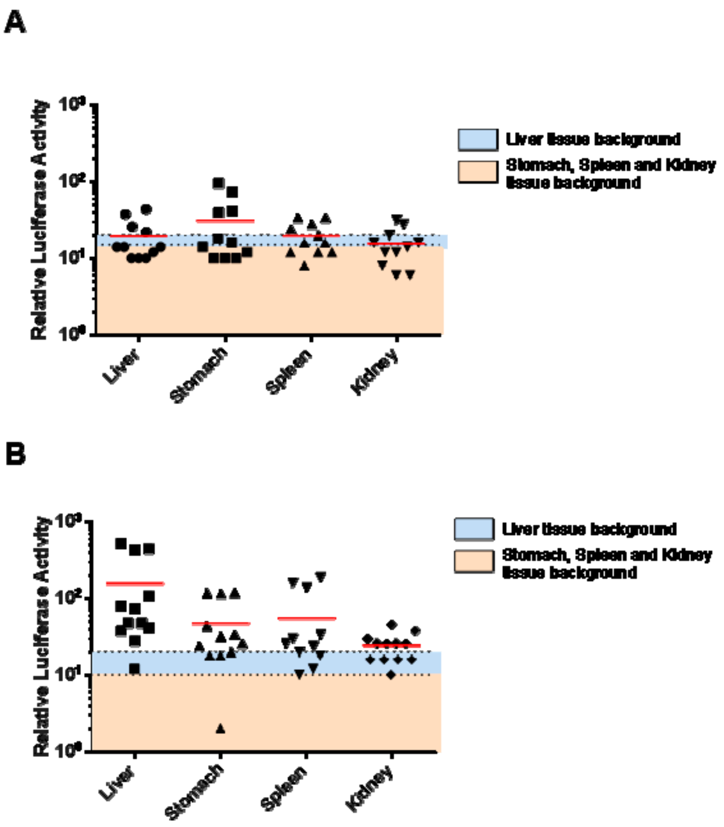

Figure S3. GBM tumour cell detection in organs of mice orthotopically implanted with U87MG-Fluc and MU20-Fluc labelled cells. Mice were implanted with U87MG-Fluc (A) or MU20-Fluc (B) glioblastoma tumours. Following euthanasia, organs were homogenised and lysed for luciferase analysis. Data represents the mean of 3 independent 5mg tissue samples per mouse, (n=4).
